# Dispersion of transposable elements and multigene families: Microstructural variation in *Characidium* (Characiformes: Crenuchidae) genomes

**DOI:** 10.1590/1678-4685-GMB-2017-0121

**Published:** 2018-07-16

**Authors:** Marcela Baer Pucci, Viviane Nogaroto, Orlando Moreira-Filho, Marcelo Ricardo Vicari

**Affiliations:** 1 Departamento de Genética e Evolução, Universidade Federal de São Carlos, São Carlos, SP, Brazil; 2 Departamento de Biologia Estrutural, Molecular e Genética, Universidade Estadual de Ponta Grossa Ponta Grossa, PR, Brazil

**Keywords:** Mobile DNA, histones, karyotype evolution, snRNA, WZ/ZZ

## Abstract

Eukaryotic genomes consist of several repetitive DNAs, including dispersed DNA sequences that move between chromosome sites, tandem repeats of DNA sequences, and multigene families. In this study, repeated sequences isolated from the genome of *Characidium gomesi* were analyzed and mapped to chromosomes in *Characidium zebra* and specimens from two populations of *C. gomesi.* The sequences were transposable elements (TEs) named retroelement of *Xiphophorus (Rex);* multigene families of *U2 small nuclear RNA (U2 snRNA);* and histones H1, H3, and H4. Sequence analyses revealed that *U2 snRNA* contains a major portion corresponding to the Tx1-type non-LTR retrotransposon *Keno,* the preferential insertion sites of which are *U2 snRNA* sequences. All histone sequences were found to be associated with TEs. *In situ* localization revealed that these DNA sequences are dispersed throughout the autosomes of the species, but they are not involved in differentiation of the specific region of the W sex chromosome in *C. gomesi.* We discuss mechanisms of TE invasion into multigene families that lead to microstructural variation in *Characidium* genomes.

## Introduction

The genomes of all studied eukaryotic species primarily consist of repetitive sequences that are dispersed or found in tandem (Sumner, 2003). Repetitive sequences were identified in fragile sites and evolutionary break point regions, promoting non-B DNA conformations and double-strand breaks, which are involved in chromosomal rearrangements (Eichler and Sankoff, 2003; Szamalek, 2005; Wells, 2007; Barros *et al*., 2017). Repetitive sequences are also responsible for a significant portion of the karyotype variations observed in many groups of organisms (Kidwell, 2002).

Dispersed DNA sequences can move between chromosome sites, with this movement occurring in the presence or absence of RNA as a transposition intermediate (Tollis and Boissinot, 2012). These mobile segments are called transposable elements (TEs) and are classified as retrotransposons (class I elements, RNA intermediates of the transposition process) or transposons (class II elements, DNA intermediates of the transposition process) (Wicker *et al.*, 2007). These mobile elements can drive genetic and genomic evolution and influence eukaryotic gene regulatory systems (Feschotte, 2008). In addition to consisting of dispersed DNA sequences, eukaryotic genomes are also enriched in tandem repeats of DNA sequences (Hardman, 1986) and groups of repeated and linked genes located at the same chromosomal region, shaping clustered but not tandemly repeated genes such as multigene families (Hentschel and Birnstiel, 1981; Heintz *et al.*, 1991).

A multigene family is described as a group of genes with similar functions and sequences that originate from a common ancestral gene (Nei and Rooney, 2005). The *U2 small nuclear RNA* (*U2 snRNA*) sequence represents a multigene family of snRNA that control premessenger RNA intron splicing (Nei and Rooney, 2005). Histone genes do not have introns, and they comprise a multigene family in which the five genes are in the same order but separated by spacer DNA (Hentschel and Birnstiel, 1981). In the rainbow trout (*Salmo gairdneri*), the histones are present in the order of *H4-H2B-H1-H2A-H3*, and they are transcribed from the same strand (Connor *et al.*, 1984).

Concerning genome diversification, fish represent an important group for studies of genetic variability. The genus *Characidium* (Characiformes: Crenuchidae) presents a diversified karyotype microstructure despite its conserved karyotype macrostructure and prevalent diploid number (2n) of 50 (Centofante *et al.*, 2001, 2003; Vicari *et al.*, 2008; Pazian *et al.*, 2013; Scacchetti *et al.*, 2015a; Pucci *et al.*, 2016; Serrano *et al.*, 2017). The *Characidium* species studied to date exhibited differences mainly in the number of ribosomal DNA sites and sex chromosomes (Pansonato-Alves *et al.*, 2010, 2011, 2014; Pucci *et al.*, 2014; Scacchetti *et al.*, 2015a, Utsunomia *et al.*, 2017), as well as an interesting dynamic of repetitive DNAs (Scacchetti *et al.*, 2015b; Pucci *et al.*, 2016).

The primary goal of this study was to perform sequence analyses and chromosome mapping of some repeated sequences isolated from the genome of *C. gomesi.* Retroelement of *Xiphophorus (Rex)* TEs were mapped to chromosomes to elucidate their possible involvement in *Characidium* karyotype evolution and diversification. The multigene families of *U2 snRNA* and histones *H1, H3,* and *H4* were also investigated through chromosome mapping and sequence analyses. Our study revealed associations between TEs and the multigene families. The obtained results will improve our understanding of the evolution and diversification of *Characidium* genomes.

## Materials and Methods

### Sampling and chromosome preparation

Individuals of the following species were collected at the indicated locations: *C. zebra* (15 specimens; Paiol Grande Stream, São Bento do Sapucaí, SP) and *C. gomesi* (nine specimens; Paiol Grande Stream, São Bento do Sapucaí, SP/five specimens; São João River, Carambeí, PR). Chromosomes for analyses were obtained using the ‘air-drying’ procedure (Bertollo *et al.*, 1978). The analyzed specimens were then deposited in the following ichthyology museums: Núcleo de Pesquisas em Limnologia, Ictiologia e Aquicultura (Nupelia), Universidade Estadual de Maringá, and Museu Nacional, Rio de Janeiro, Brazil, voucher numbers (NUP 14577-14580; MNRJ 29183). The processing was performed in accordance with the Ethical Committee on Animal Use (CEUA 29/2016) of the Universidade Estadual de Ponta Grossa and current Brazilian legislation. Chromosome preparations were subjected to conventional Giemsa staining to determine 2n and the chromosome formula.

### Sequence isolation

The analyzed sequences were synthesized by polymerase chain reaction (PCR) using genomic DNA from *C. gomesi* (São João River population), and the reaction mixtures consisted of 100-200 ng of genomic DNA, 0.04-0.2 μM primers, 0.04-0.16 mMdNTPs, 1 U of *Taq* DNA Polymerase (Invitrogen, Waltham, MA, USA), and 1.5 mM MgCl_2_ in a 1 reaction buffer (200 mM Tris, pH 8.4, 500 mM KCl). The specific PCR mixtures and primers sequences are summarized in Table S1. The PCR conditions were as follows: (i) *Rex1* and *Rex3* probes: 95 °C for 5 min, 35 cycles of 95 °C for 1 min, 55 °C for 40 s and 72 °C for 2 min, and a final extension at 72 °C for 5 min; (ii) *U2 snRNA* probe: 95 °C for 45 s, 30 cycles of 95 °C for 45 s, 52 °C for 45 s and 72 °C for 80 s, and a final extension at 72 °C for 7 min; and (iii) histones H1, H3, and H4: 95 °C for 5 min, 30 cycles of 95 °C for 30 s, 52 °C for 45 s and 72 °C for 80 s, and a final extension at 72 °C for 7 min.

### TEs and multigene family sequences: Sequencing and analyses

After the amplification reactions, the PCR products were purified using the GenElute PCR Clean-Up Kit (Sigma Aldrich, St Louis, MO, USA). *Rex1* and *Rex3* sequences were cloned using pGEM®-T Easy Vector Systems (Promega, Madison, WI, USA). The obtained clones were sequenced using an ABI-PRISM Genetic Analyzer (Applied Biosystems, Carlsbad, CA, USA). The sequences were edited and analyzed using Geneious 7.1.3 software (Kearse *et al.*, 2012), and their identities were confirmed using the CENSOR tool for repeated sequences (Girinst) (Kohany *et al.*, 2006) and BLAST*n* (NCBI). Finally, the sequences were deposited in GenBank (Table S2).

### Probe preparation

The sequences of *Rex3, U2 snRNA,* and histones H1 and H4 were labeled with digoxigenin via nick translation using DIG-Nick Translation Mix (Roche Applied Science, Penzberg, Germany), and those of *Rex1* and H3 were bio-tinylated using Biotin-Nick Translation Mix (Roche Applied Science). A *C. gomesi* W-specific chromosome probe was constructed as described by Machado *et al.* (2011), labeled with digoxigenin 11-dUTP (Roche Applied Science), and used in fluorescence *in situ* hybridization (FISH) to identify sex chromosomes in the karyotypes.

### FISH

Chromosome spreads were subjected to FISH using the constructed probes. FISH was performed under a high stringency of approximately 76% (2.5 ng/μL of each probe, 50% formamide, 2 SSC, 10% dextran sulfate, pH 7.0–7.2, 37 °C overnight) following the general procedure described by Pinkel *et al.* (1986). Signal detection was performed using an anti-streptavidin antibody conjugated to Alexa Fluor 488 (Molecular Probes, Eugene, OR, USA) and an anti-digoxigenin antibody conjugated to rhodamine (Roche Applied Science). Chromosomes were counterstained with 4'6-diamidino-2-phenylindole (0.2 μg/mL) in Vectashield mounting medium (Vector Laboratories, Burlingame, CA, USA) and observed under an epifluorescence microscope.

### Karyotype analysis

Approximately 20 metaphases were analyzed for each species, and karyotypes were determined from the highest-quality images. Chromosomes were classified as metacentric, submetacentric, subtelocentric, or acrocentric according to the arm ratio (Levan *et al.*, 1964) and arranged by decreasing size in the karyotypes.

## Results

### Analyses of partial sequences of TEs and multigene families

**Figure 1:**
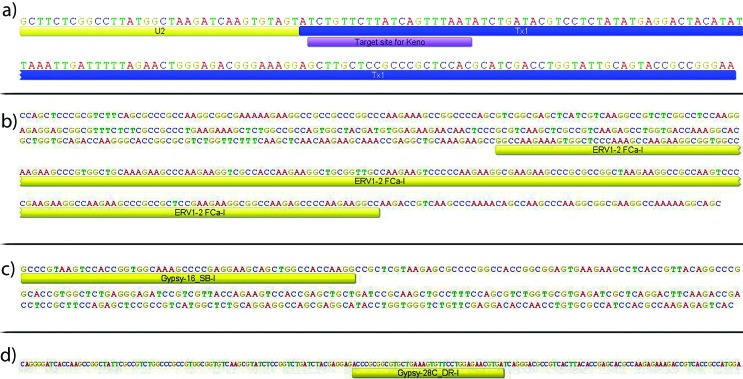
Partial sequences of multigene families isolated from *C. gomesi* genome, with TE insertion. (a) Partial sequence of the *U2 snRNA* gene (yellow), associated with its specific U2-target *Keno* TE (blue); histone partial sequences, with the internal portion of TEs; (b) H1 with retrotransposon *ERV1* (yellow); (c) H3 with retrotransposon *Gypsy* (yellow); (d) H4 with retrotransposon *Gypsy* (yellow).

The partial sequences of *Rex1*, *Rex3*, *U2 snRNA*, and the H1, H3, and H4 genes were isolated from the genomes of *C. gomesi* and *C. zebra*, and consensus sequence of each gene was constructed (Table S2). When analyzed using the CENSOR tool, the multigene family sequences displayed high proportions of retrotransposon sequences as follows: *U2 snRNA* contained the Tx1-type element called *Keno-1_SSa* (Figure 1a); H1 contained an internal portion (217 bp) of an *ERV1-*type endogenous retrovirus sequence (Figure 1b); H3 displayed an internal portion (52 bp) of the LTR retrotransposon *Gypsy* (Figure 1c), although chromosome mapping of this sequence only revealed the main H3 histone clusters with no evidence of dispersed clusters; and H4 contained an internal portion (37 bp) of the LTR retro-transposon *Gypsy* (Figure 1d).

### Cytogenetics of *Characidium*

**Figure 2:**
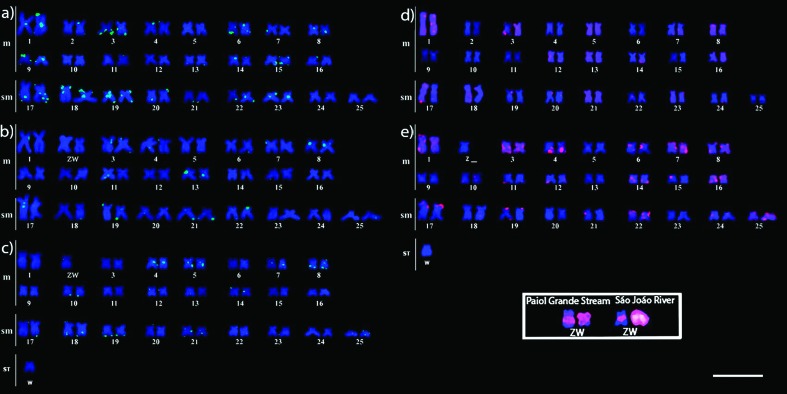
Karyotypes of *Characidium* females subjected to fluorescence *in situ* hybridization (FISH) with TE probes. (a) *C. zebra,* (b) *C. gomesi* (PG), (c) *C. gomesi* (SJ); (d) *C. zebra,* (e) *C. gomesi* (SJ). The *Rex3* probe did not show any hybridization signals in *C. gomesi* (PG) chromosomes (not shown). The W and Z sex chromosomes of *C. gomesi* females are highlighted in the box. PG, Paiol Grande Stream population; SJ, São João River population. Scale bar, 10 μm.

The studied species presented a 2n of 50, and these chromosomes have been cytogenetically described by Machado *et al.* (2011) and Pucci *et al.* (2014). Karyotype formulae were organized as 32 metacentric + 18 submeta-centric, excluding females of *C. gomesi* (São João River population), which were organized as 31 metacentric + 18 submetacentric + 1 subtelocentric. The fundamental number of chromosome arms was 100 in all studied species/populations. No differentiated sex chromosomes were found in the *C. zebra* population. The *C. gomesi* W-specific probe revealed sex chromosomes as metacentric pair 2 in *C. gomesi* from the Paiol Grande Stream population and metacentric Z position 2 and subtelocentric W in *C. gomesi* from the São João River population (Figure 2, Z and W chromosomes are highlighted in the box).

### Chromosome mapping of Rex1 and Rex3 on *Characidium* chromosomes

The non-LTR retrotransposons *Rex1* and *Rex3* in *C. zebra* and *C. gomesi* were observed in a few chromosomes (Figure 2a–e). In *C. zebra*, *Rex1* displayed more prominent hybridization signals in metacentric pair 3 and submeta-centric pairs 18 and 19 (Figure 2a). In *C. gomesi* from the Paiol Grande Stream population, *Rex1* exhibited strong signals in metacentric pairs 8 and 13 (Figure 2b). In *C. gomesi* from the São João River population, *Rex1* exhibited clear marks in metacentric pairs 4, 5, and 8 and submetacentric pair 19 (Figure 2c). However, *Rex1* did not display clear marks in the Z and W chromosomes either *C. gomesi* population (Figure 2b–c). In *C. zebra*, *Rex3* exhibited convincing hybridization signals in metacentric pairs 1, 3, and 8 and submetacentric pair 17 (Figure 2d). In *C. gomesi* from the São João River population, *Rex3* displayed signals in metacentric pairs 1, 3, 4, 6, 7, 8, 14, and 16 and submetacentric pairs 17, 22, and 25 (Figure 2e). *Rex3* did not hybridize with the Z and W chromosomes of *C. gomesi* from the São João River population (Figure 2e), nor did it exhibit hybridization signals in any chromosome of *C. gomesi* from the Paiol Grande Stream population (data not shown).

### Chromosome mapping of multigene families U2 snRNA and the H1, H3, and H4 genes on *Characidium* chromosomes

**Figure 3:**
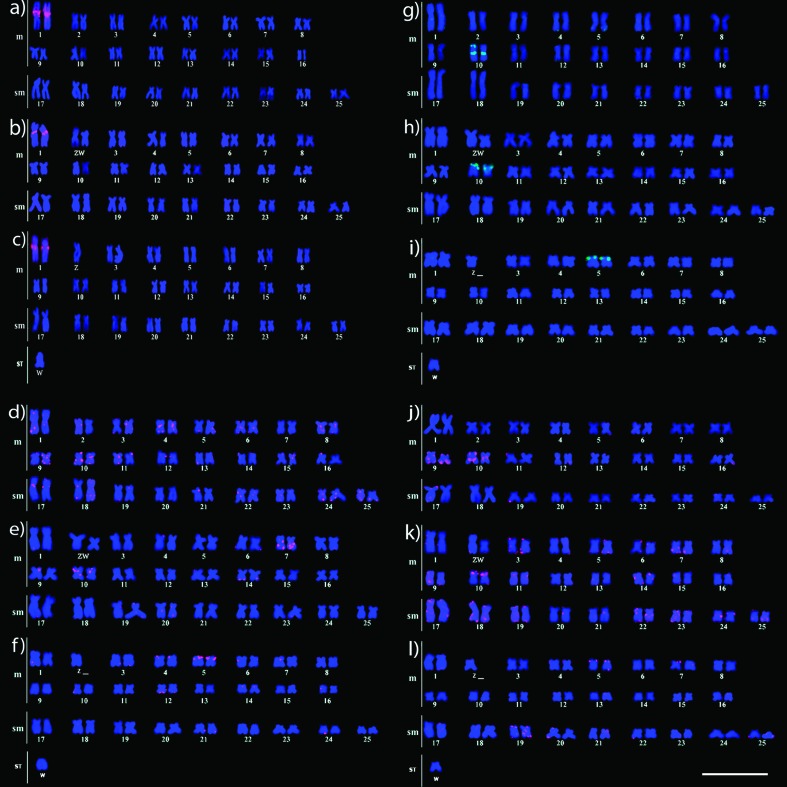
Karyotypes of *Characidium* females subjected to fluorescence *in situ* hybridization (FISH) with multigene family probes. (a) *C. zebra,* (b) *C. gomesi* (PG), (c) *C. gomesi* (SJ); H1 (d) *C. zebra,* (e) *C. gomesi* (PG), (f) *C. gomesi* (SJ); (g) *C. zebra,* (h) *C. gomesi* (PG), (i) *C. gomesi* (SJ); (j) *C. zebra,* (k) *C. gomesi* (PG), (l) *C. gomesi* (SJ). PG, Paiol Grande Stream population; SJ, São João River population. Scale bar, 10 μm.

The *U2 snRNA* probe displayed a single cluster of hybridization signals in the pericentromeric region of meta-centric pair 1 in all analyzed species, with no additional dispersed sites detected (Figure 3a–c).

The H1 histone gene probe displayed primary clusters of hybridization signals in the pericentromeric region and short arm of one chromosome of metacentric pair 10, whereas only one cluster was found in the pericentromeric region of the other chromosome in pair 10 of *C. zebra* (Figure 3d) and metacentric pair 10 of *C. gomesi* (Paiol Grande Stream population). An additional cluster was noted in pair 7 of *C. gomesi* from the Paiol Grande Stream population (Figure 3e) and metacentric pair 5 of *C. gomesi* from the São João River population (Figure 3f). In addition, each species exhibited weak additional signals in several other autosomes (Figure 3d–f).

The H3 gene probe displayed primary clusters of hybridization signals in the pericentromeric region and short arm of one chromosome of metacentric pair 10 and one cluster in the pericentromeric region of the other chromosome in pair 10 of *C. zebra* (Figure 3g), the short arm of metacentric pair 10 of *C. gomesi* from the Paiol Grande Stream population (Figure 3h). One cluster was also found in the short arm of metacentric pair 5 of *C. gomesi* from the São João River population (Figure 3i).

The H4 gene probe revealed primary clusters of hybridization signals in the pericentromeric region and short arm of one chromosome in metacentric pair 10 and one cluster in the pericentromeric region of the other chromosome in pair 10 of *C. zebra*, as well as additional marks in metacentric pair 9 (Figure 3j) and the short arm of meta-centric pair 10 of *C. gomesi* from the Paiol Grande Stream population (Figure 3k) and a weak signal in metacentric pair 5 of *C. gomesi* from the São João River population (Figure 3l). Marks were also noted in some autosomes of both populations of *C. gomesi* (Figure 3k–l).

## Discussion

### Distribution of Rex1 and Rex3 on *Characidium* chromosomes

*Rex* elements are non-LTR retrotransposons (Wicker *et al.*, 2007) that are extensively distributed through fish genomes (Ozouf-Costaz *et al.*, 2004; Ferreira *et al.*, 2010; Borba *et al.*, 2013; Schneider *et al.*, 2013; Yano *et al.*, 2014; Sene *et al.*, 2015; Pinheiro *et al.*, 2016), in addition to those of other species. *Rex1* and *Rex3* are significant sequences in the organization and evolution of the genomes in most of the aforementioned species, as indicated by evident hybridization signals and prominent amounts of these sequences. In this analysis, *Rex1* and *Rex3* elements were dispersed in small clusters throughout the chromosomes, and they did not display significant chromosome reorganization between *Characidium* species.

Concerning the distribution of *Rex1* and *Rex3* in the sex chromosomes, no hybridization sites were identified in the Z and W sex chromosomes of *Characidium*, whereas these elements are involved in sex chromosome evolution in other species. In particular, *Rex3* was detected in the Y chromosome of *Chionodraco hamatus* (Ozouf-Costaz *et al.*, 2004) and X chromosome of *Eigenmannia* (Sene *et al.*, 2015); *Rex1* and *Rex3* were found in the W chromosome of *Leporinus* (Borba *et al.*, 2013); and *Rex1*, *Rex3*, and *Rex6* were identified in the Z and W chromosomes of *Triportheus* (Yano *et al.*, 2014). The *Rex1* and *Rex3* elements analyzed in the *Characidium* genome emerged in the ancestral species *C. zebra*. However, these elements did not exhibit high transposition rates, presenting only small clusters in some autosomes in all analyzed species. Moreover, the *Rex3* element was not identified in the genome of *C. gomesi* from the Paiol Grande Stream population. Natural selection may minimize the transposition rate, promoting vertical inactivation (Lohe *et al.*, 1995), which could be true for *Rex* elements in *Characidium*. Another possible explanation for the low transposition rate could be stochastic loss, in which the element is gradually removed from the genome, as observed for *mariner-*like elements in the *Drosophila melanogaster* species complex (Lohe *et al.*, 1995) and probably for *Rex3* in *C. gomesi* from the São João River population.

### Multigene families and TE insertions

Chromosome mapping of *U2 snRNA* revealed localized clusters in the first metacentric pair in all studied species. In fact, the distribution pattern of *U2 snRNA* is highly conserved for *Characidium*, as described by Scacchetti *et al.* (2015a), with only some exceptions such as *Characidium* sp. aff. *C. vidali*, *Characidium* sp. 1 (Scacchetti *et al.*, 2015a), and *C. alipioi* (Serrano *et al.*, 2017). *U2 snRNA* sequences appear to be conserved in other species, and co-localization and linkage between U2 genes and ribosomal sites has been reported (Cross and Rebordinos, 2005; Manchado *et al.*, 2006; Úbeda-Manzanaro *et al.*, 2010; Scacchetti *et al.*, 2015a). Despite the presence of conserved clusters, sequence analyses of *U2 snRNA* using the CENSOR tool revealed a major portion corresponding to the Tx1-type non-LTR retrotransposon *Keno-1_SSa* (Kohany *et al.*, 2006). There are several sequence-specific families in the Tx element group, and *Keno* is specific for *U2 snRNA* (Kojima and Fujiwara, 2004). Insertion of the *Keno* element occurs at a specific site 37 nu-cleotides downstream of *U2 snRNA*, and its insertion destroys the target (Kojima and Fujiwara, 2004). The *Keno-1_SSa* (Kohany *et al.*, 2006) element found in the *U2 snRNA* sequence of *Characidium* is classified as *KenoDr1* because the specific 3’ target sequence (TCTGTTCTTATCAGTTTAAT) localized 37 nucleotides downstream of *U2 snRNA* (Kojima and Fujiwara, 2004; Kojima and Jurka, 2015). Despite the TE insertion, the *U2 snRNA* sequence did not exhibit additional clusters.

*In situ* localization for the H1, H3, and H4 sequences revealed primary clusters in metacentric pair 10 of *C. zebra* and *C. gomesi* from the Paiol Grande Stream population as well as metacentric pair 5 of *C. gomesi* from the São João River population. Additional hybridization signals for H1 and H4 were dispersed through the autosomes of the three populations, although not in the sex chromosomes. Chromosomal rearrangement and the absence of gene flow resulted in the differentiated karyotype of *C. gomesi* from the São João River population, which exhibited primary clusters of H1, H3, and H4 in metacentric pair 5 (translocation) and subtelocentric sex chromosome W (inversion). The sites of H3 were also localized to metacentric pair 10 in *C. alipioi* (Serrano *et al.*, 2017), albeit in the long arms, pointing to the occurrence of rearrangements involving these chromosomes. Our analyses of the histone sequences also revealed LTR retrotransposon (Wicker *et al.*, 2007) insertions of *ERV1* (H1) and *Gypsy* (H3 and H4). The LTR retrotransposon *Gypsy* inserted in the H3 sequence was not involved in the spread of this sequence throughout the genome. Additional clusters of H1 and H4 are probably due to the involvement of TEs. Hence, the major force leading to chromosomal spread of the H1 and H4 sequences in the *Characidium* karyotypes were probably a consequence of hitchhiking by H1 and H4 with the mobile elements-mediated transposition events. However, these additional H1 and H4 chromosomal marks could represent the *Gypsy* and *ERV1* TE sequences alone without the histone genes adjacent to them.

Insertion of a TE inside or around a gene can alter its expression considerably, increasing or decreasing its expression when the insertion occurs in promoter regions, (Finnegan, 1989), or block gene expression by disrupting normal gene function (Chuong *et al.*, 2016). However, it is difficult at present to determine the consequences of retrotransposon insertions in *U2 snRNA* and the H3 gene of *Characidium*, as they are essential for cellular function.

Our results illustrated that the *Characidium* genome is dynamic concerning TEs. However, these TEs did not promote deep chromosomal reorganization of the *Characidium* karyotypes, nor were they involved in differentiation of the specific W sex chromosome region in *C. gomesi*. It is therefore desirable to identify and map other TEs in the *Characidium* genome to improve our understanding of karyotype and sex chromosome evolution in this fish genus. However, the results presented in this study will enable the detection of innumerous TE insertions/transpositions generating microstructural variation in *Characidium* genomes, including some TE invasions in gene families.

## Supplementary material

The following online material is available for this article:

Table S1- PCR reaction mixture for the synthesis of probes used in this work.

Table S2 - Partial sequences isolated from the *Characidium* species/population genome.
