# Supplementary material for: Dispersion of transposable elements and multigene families: Microstructural variation in Characidium (Characiformes: Crenuchidae) genomes
Source: Genet Mol Biol. 2018 Jul 16;41(3):585–92. doi: 10.1590/1678-4685-GMB-2017-0121 (PMC6136364; doi:10.1590/1678-4685-GMB-2017-0121)
Supplement: Supplementary file 1 [file 1415-4757-GMB-1678-4685-GMB-2017-0121-s001.pdf]

Supplementary Material to: "Dispersion of transposable elements and multigene families: Microstructural variation in *Characidium* (Characiformes: Crenuchidae) genomes"

Table S1 - PCR reaction mixture for the synthesis of probes used in this work.

Probe/ Reaction Mix	Primer Forward/Reverse	Vol (µl)	DNA (ng)	$MgCl_2$ (mM)	Primer (µM)	dNTP (mM)	*Taq* pol* (U/µl)
Rex1	5'- TTCTCCAGTGCCTTCAACACC-3'	25	200	1,5	0,2	0,04	1
	5'- TCCCTCAGCAGAAAGAGTCTGCTC-3'						
	(Volff *et al.,* 2000)						
Rex3	5'-CGGTGAAAAGGGCAGCCCTG-3'	25	200	1,5	0,2	0,04	1
	5'-TGGCAGACNGGGGTGGTGGT-3'						
	(Volff *et al.,* 1999)						
U2	5'- ATCGCTTCTCGGCCTTATG -3'	25	100	1,5	0,04	0,8	1
snRNA	3'- TCCCGGCGGTACTGCAATA -5'						
	(Bueno *et al.,* 2013)						
H1	5'-ATGGCAGAARYCGCMCCAGC-3'	25	100	1,5	0,2	0,16	1
	5'-TACTTCTTCTTGGGSGCTGC-3'						
	(Hashimoto *et al.,* 2011)						
H3	5'-ATGGCTCGTACCAAGCAGACVGC-3'	25	100	1,5	0,2	0,16	1
	5'-ATATCCTTRGGCATRATRGTGAC-3'						
	(Colgan *et al.,* 1998)						
H4	5'-TSCGIGAYAACATYCAGGGIATCAC-3'	25	100	1,5	0,2	0,16	1
	5'-CKYTTIAGIGCRTAIACCACRTCCAT-3'						
	(Pineau *et al.,* 2005)						

**Taq* polymerase

References

Bueno D, Palacios-Gimenez OM and Cabral-de-Mello DC (2013) Chromosomal mapping of repetitive DNAs in the grasshopper *Abracris flavolineata* reveal possible ancestry of the B chromosome and H3 histone spreading. PLoS One 8:e66532.

Colgan D, McLauchlan A, Wilson G and Livingston S (1998) Histone H3 and U2 snRNA DNA sequences and arthropod molecular evolution. Aust J Zool 46:419-437.

Hashimoto DT, Ferguson-Smith MA, Rens W, Foresti F and Porto- Foresti F (2011) Chromosome mapping of H1 histone and 5S rRNA gene clusters in three species of *Astyanax* (Teleostei, Characidae). Cytogenet Genome Res 134:64-71.

Pineau P, Henry M, Suspène R, Marchio A, Dettai A, Debruyne R, Petit T, Lécu A, Moisson P, Dejean A *et al.* (2005) A universal primer set for PCR amplification of nuclear histone H4 genes from all animal species. Mol Biol Evol 22:582-588.

Volf JN, Körting C, Sweeney K and Schart M (1999) The Non-LTR retrotransposon Rex3 from the fish *Xiphophorus* is widespread among teleosts. Mol Biol Evol 16:1427-1438.

Volff JN, Korting K and Schartl M (2000) Multiple lineages of the non-LTR retrotransposon *Rex1* with varying success in invading fish genomes. Mol Biol Evol 17:1673-1684.
