# Supplementary material for: Dispersion of transposable elements and multigene families: Microstructural variation in Characidium (Characiformes: Crenuchidae) genomes
Source: Genet Mol Biol. 2018 Jul 16;41(3):585–92. doi: 10.1590/1678-4685-GMB-2017-0121 (PMC6136364; doi:10.1590/1678-4685-GMB-2017-0121)
Supplement: Supplementary file 2 [file 1415-4757-GMB-1678-4685-GMB-2017-0121-s002.pdf]

Supplementary Material to: “Dispersion of transposable elements and multigene families: Microstructural variation in *Characidium* (Characiformes: Crenuchidae) genomes”

Table S2 - Partial sequences isolated from the *Characidium* species/population genome.

Partial sequence	Length (base pair)	GenBank accession number	Species/Population
Rex1 *	499	KY566212	*C. gomesi* (São João)
Rex1	479	MG027999	*C. gomesi* (Paiol Grande)
Rex1	492	MG028004	*C. zebra* (Paiol Grande)
*Rex3**	496	KY566213	*C. gomesi* (São João)
Rex3	465	MG028000	*C. gomesi* (Paiol Grande)
Rex3	466	MG028005	*C. zebra* (Paiol Grande)
U2 snRNA gene*	175	KY566214	*C. gomesi* (São João)
U2 snRNA gene	149	MG028001	*C. gomesi* (Paiol Grande)
U2 snRNA gene	136	MG028006	*C. zebra* (Paiol Grande)
Histone H1*	587	KY566215	*C. gomesi* (São João)
Histone H3*	335	KY566216	*C. gomesi* (São João)
Histone H3	257	MG028002	*C. gomesi* (Paiol Grande)
Histone H3	335	MG028007	*C. zebra* (Paiol Grande)
Histone H4*	176	KY566217	*C. gomesi* (São João)
Histone H4	134	MG028003	*C. gomesi* (Paiol Grande)
Histone H4	123	MG028008	*C. zebra* (Paiol Grande)

*Sequences used as probes in the fluorescence *in situ* hybridization (Figures 2 and 3) and described in Figure 1.
